# Gene model for the ortholog of *Dsor1* in *Drosophila persimilis*

**DOI:** 10.17912/micropub.biology.000853

**Published:** 2024-12-08

**Authors:** Alyssa C. Koehler, Emma Seay, Hannah Ewing, Zachary Mearse, Ana Maria Rocha de Almeida, Sara Cline, Jamie Siders, Lindsey J. Long, Chinmay P. Rele, Laura K. Reed

**Affiliations:** 1 University of Alabama, Tuscaloosa, AL US; 2 Oklahoma Christian University, Edmond, OK USA; 3 California State University-East Bay, Hayward, CA USA; 4 Athens State University, Athens, AL USA; 5 Ohio Northern University, Ada, OH USA; 6 Biological Sciences, University of Alabama, Tuscaloosa, AL US

## Abstract

Gene model for the ortholog of Downstream of raf1 ( *Dsor1* ) in the May 2011 (Broad dper_caf1/DperCAF1) Genome Assembly (GenBank Accession: GCA_000005195.1 ) of *Drosophila persimilis* . This ortholog was characterized as part of a developing dataset to study the evolution of the Insulin/insulin-like growth factor signaling pathway (IIS) across the genus *Drosophila* using the Genomics Education Partnership gene annotation protocol for Course-based Undergraduate Research Experiences.

**Figure 1. Genomic neighborhood and gene model for Dsor1 in Drosophila persimilis:**
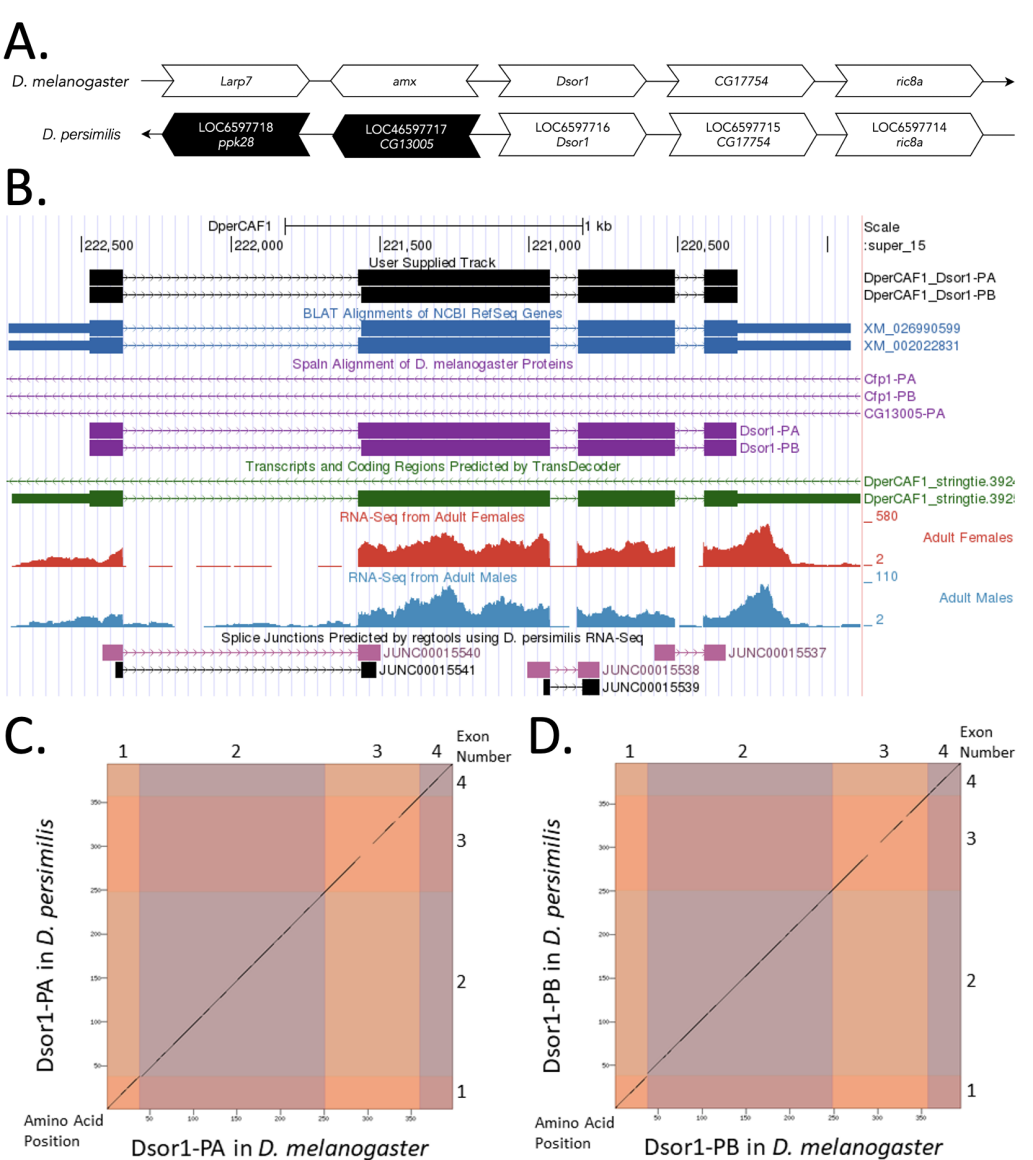
**
(A) Synteny comparison of the genomic neighborhoods for *Dsor1* in *Drosophila melanogaster* and *D. persimilis* .** Thin underlying arrows indicate the DNA strand within which the target gene– *Dsor1* –is located in *D. melanogaster* (top) and *D. persimilis* (bottom). The thin arrow pointing to the right indicates that *Dsor1* is on the positive (+) strand in *D. melanogaster* , and the thin arrow pointing to the left indicates that *Dsor1* is on the negative (-) strand in *D. persimilis* . The wide gene arrows pointing in the same direction as *Dsor1* are on the same strand relative to the thin underlying arrows, while wide gene arrows pointing in the opposite direction of *Dsor1* are on the opposite strand relative to the thin underlying arrows. White gene arrows in *D. persimilis* indicate orthology to the corresponding gene in *D. melanogaster* , while black gene arrows indicate non-orthology. Gene symbols given in the *D. persimilis* gene arrows indicate the orthologous gene in *D. melanogaster* , while the locus identifiers are specific to *D. persimilis* . **(B) Gene Model in GEP UCSC Track Data Hub (Raney *et al* ., 2014).** The coding-regions of *Dsor1* in *D. persimilis* are displayed in the User Supplied Track (black); coding CDSs are depicted by thick rectangles and introns by thin lines with arrows indicating the direction of transcription. Subsequent evidence tracks include BLAT Alignments of NCBI RefSeq Genes (dark blue, alignment of Ref-Seq genes for *D. persimilis* ), Spaln of *D. melanogaster* Proteins (purple, alignment of Ref-Seq proteins from *D. melanogaster* ), Transcripts and Coding Regions Predicted by TransDecoder (dark green), RNA-Seq from Adult Females and Adult Males (red and light blue, respectively; alignment of Illumina RNA-Seq reads from *D. persimilis* ), and Splice Junctions Predicted by regtools using *D. persimilis* RNA-Seq (Yang *et al* ., 2018; PRJNA388952 ). Splice junctions shown in pink have read-depths of 100-499, while the black junctions have a read-depths <10. **(C) Dot Plot of Dsor1-PA in *D. melanogaster* ( *x* -axis) vs. the orthologous peptide in *D. persimilis* ( *y* -axis).** Amino acid number is indicated along the left and bottom; coding-CDS number is indicated along the top and right, and CDSs are also highlighted with alternating colors. **(D) Dot Plot of Dsor1-PB in *D. melanogaster* ( *x* -axis) vs. the orthologous peptide in *D. persimilis* ( *y* -axis)
**

## Description

**Table:** 

* This article reports a predicted gene model generated by undergraduate work using a structured gene model annotation protocol defined by the Genomics Education Partnership (GEP; thegep.org) for Course-based Undergraduate Research Experience (CURE). The following information may be repeated in other articles submitted by participants using the same GEP CURE protocol for annotating Drosophila species orthologs of Drosophila melanogaster genes in the insulin signaling pathway. * "In this GEP CURE protocol students use web-based tools to manually annotate genes in non-model *Drosophila* species based on orthology to genes in the well-annotated model organism fruitfly *Drosophila melanogaster* . The GEP uses web-based tools to allow undergraduates to participate in course-based research by generating manual annotations of genes in non-model species (Rele et al., 2023) . Computational-based gene predictions in any organism are often improved by careful manual annotation and curation, allowing for more accurate analyses of gene and genome evolution (Mudge and Harrow 2016; Tello-Ruiz et al., 2019) . These models of orthologous genes across species, such as the one presented here, then provide a reliable basis for further evolutionary genomic analyses when made available to the scientific community.” (Myers et al., 2024) . “The particular gene ortholog described here was characterized as part of a developing dataset to study the evolution of the Insulin/insulin-like growth factor signaling pathway (IIS) across the genus *Drosophila* . The Insulin/insulin-like growth factor signaling pathway (IIS) is a highly conserved signaling pathway in animals and is central to mediating organismal responses to nutrients (Hietakangas and Cohen 2009; Grewal 2009) .” (Myers et al., 2024) .

*Downstream of raf1* ( *Dsor1* ), a core component of the insulin signaling pathway, encodes a serine/threonine kinase that phosphorylates the mitogen-activated protein (MAP) kinase, Rolled (Rl) in *Drosophila,* and is therefore referred to as a MAP kinase kinase (MAPKK) (Lu et al., 1994; Oellers et al., 1996) . Activated by the product of the *Raf* oncogene ( *Raf* ), Dsor1 acts downstream of receptor tyrosine kinases, including Torso, Epidermal Growth Factor Receptor, and Sevenless in *Drosophila* (Tsuda et al., 1993; Lim et al., 1997; Goyal et al., 2017) .

We propose a gene model for the *D. persimilis* ortholog of the *D. melanogaster* *Downstream of raf1* ( *Dsor1* ) gene. The genomic region of the ortholog corresponds to the uncharacterized protein LOC6597716 (RefSeq accession XP_002022867.1 ) in the Dper_CAF1 Genome Assembly of *D. persimilis* (GenBank Accession: GCA_000005195.1 - Drosophila 12 Genomes Consortium et al., 2007; PRJNA388952 ). This model is based on RNA-Seq data from *D. persimilis* (Yang et al., 2018; PRJNA388952 ) and *Dsor1* in *D. melanogaster* using FlyBase release FB2022_04 ( GCA_000001215.4 ; Larkin et al., 2021; Gramates et al., 2022; Jenkins et al., 2022).

*D. persimilis* is part of the *pseudoobscura* species subgroup within the *obscura* species group in the subgenus *Sophophora* of the genus *Drosophila* (Sturtevant 1942; Buzzati-Traverso and Scossiroli 1955) . It was first described by Dobzhansky and Epling (1944). The *pseudoobscura* species subgroup is endemic to the western hemisphere, where *D. persimilis* is found in the Pacific Northwest, sympatric with its sibling species *D. pseudoobscura* (Markow and O'Grady 2005). Larvae of *D. persimilis* have been found feeding in sap fluxes from some oak species in California (Carson 1951) , however its ecology is not fully elucidated (Powell 1997) .

***Synteny***

The target gene, *Dsor1 ,* occurs on chromosome X in *D. melanogaster* and is flanked upstream by *Larp7* and almondex ( *amx* ) and downstream by *CG17754* and *ric8a* . The *tblastn* search of *D. melanogaster* Dsor1-PA (query) against the *D. persimilis* (GenBank Accession: GCA_000005195.1 ) Genome Assembly (database) placed the putative ortholog of *Dsor1* within scaffold scaffold_15 ( CH479194.1 ) at locus LOC6597716 ( XP_002022867.1 )— with an E-value of 0.0 and a percent identity of 95.71%. Furthermore, the putative ortholog is flanked upstream by LOC6597718 ( XP_002022869.2 ) and LOC6597717 ( XP_002022868.2 ), which correspond to pickpocket 28 ( *ppk28* ) and *CG13005* in *D. melanogaster* (E-value: 0.0 and 0.0; identity: 80.86% and 69.37%, respectively, as determined by *blastp* ; Figure 1A, A ltschul et al *.* , 1990). In *D. melanogaster ,* *ppk28* and *CG13005* also occur on the X-chromosome (Muller A), but at a different relative location. The putative ortholog of *Dsor1* is flanked downstream by LOC6597715 ( XP_026846396.1 ) and LOC6597714 ( XP_002022865.2 ), which correspond to *CG17754* and *ric8a* in *D. melanogaster* (E-value: 0.0 and 0.0; identity: 96.18% and 83.68%, respectively, as determined by *blastp* ). The putative ortholog assignment for *Dsor1* in *D. persimilis* is supported by the following evidence: Although the genes upstream of the *Dsor1* ortholog are not orthologous to the genes at the same locus in *D. melanogaster* , the downstream local synteny is completely conserved, supported by E-values and percent identities, so we conclude that LOC6597716 is the correct ortholog of *Dsor1* in *D. persimilis* ( Figure 1A ).

***Protein Model***

Consistent with the *blastp* search result which shows 95.71% identity between *D. melanogaster* Dsor1-PA and the *D. persimilis* gene model as well as the low sensitivity parameters used to generate the dot plot (i.e., word size = 3; neighborhood threshold = 11), the dot plot features very few gaps along the diagonal, indicating significant conservation between the two protein sequences. *Dsor1* in *D. persimilis* has two unique protein-coding isoforms (Dsor1-PA and Dsor1-PB; Figure 1B ). mRNA isoforms *Dsor1-RA* and *Dsor1-RB* contain four CDSs each. The two isoforms differ in the splice acceptor site preceding their second exon, with *Dsor1-RA* having a slightly longer second exon. The splice junction for *Dsor1-RA* has strong support with a read depth of >100, while the *Dsor1-RB* isoform specific splice junction has weaker support (<10 reads). Despite the relatively low RNAseq support for the *Dsor1-RB* isoform, which is derived from just two adult RNAseq samples, the conservation of the protein coding sequence and presence of a viable alternative splice site (AG) suggests that *Dsor1-RB* is likely to be present in *D. persimilis* as well. Further, our protocol assumes conservation of gene/isoform structure relative to *D. melanogaster* in the absence of definitive counter evidence (Rele et al., 2023) . Relative to the ortholog in *D. melanogaster* , the CDS number and isoform count are conserved. The sequence of Dsor1-PA in *D. persimilis* has 95.71% identity (E-value: 0.0) with the protein-coding isoform Dsor1-PA in *D. melanogaster* , as determined by *blastp* ( Figure 1C ). Coordinates of this curated gene model are stored by NCBI at GenBank/BankIt (accessions BK064411 and BK064412 ). These data are also archived in the CaltechDATA repository (see “Extended Data” section below).

## Methods

Detailed methods including algorithms, database versions, and citations for the complete annotation process can be found in Rele et al. (2023). Briefly, students use the GEP instance of the UCSC Genome Browser v.435 (https://gander.wustl.edu ; Kent WJ et al., 2002; Navarro Gonzalez et al., 2021) to examine the genomic neighborhood of their reference IIS gene in the *D. melanogaster* genome assembly (Aug. 2014; BDGP Release 6 + ISO1 MT/dm6). Students then retrieve the protein sequence for the *D. melanogaster* target gene for a given isoform and run it using *tblastn* against their target *Drosophila* species genome assembly ( *D. persimilis* (GenBank Accession: GCA_000005195.1 )) on the NCBI BLAST server (https://blast.ncbi.nlm.nih.gov/Blast.cgi, Altschul et al., 1990) to identify potential orthologs. To validate the potential ortholog, students compare the local genomic neighborhood of their potential ortholog with the genomic neighborhood of their reference gene in *D. melanogaster* . This local synteny analysis includes at minimum the two upstream and downstream genes relative to their putative ortholog. They also explore other sets of genomic evidence using multiple alignment tracks in the Genome Browser, including BLAT alignments of RefSeq Genes, Spaln alignment of *D. melanogaster* proteins, multiple gene prediction tracks (e.g., GeMoMa, Geneid, Augustus), and modENCODE RNA-Seq from the target species. Genomic structure information (e.g., CDSs, CDS number and boundaries, number of isoforms) for the *D. melanogaster* reference gene is retrieved through the Gene Record Finder (https://gander.wustl.edu/~wilson/dmelgenerecord/index.html; Rele et al *.,* 2023). Approximate splice sites within the target gene are determined using *tblastn* using the CDSs from the *D. melanogaste* r reference gene. Coordinates of CDSs are then refined by examining aligned modENCODE RNA-Seq data, and by applying paradigms of molecular biology such as identifying canonical splice site sequences and ensuring the maintenance of an open reading frame across hypothesized splice sites. Students then confirm the biological validity of their target gene model using the Gene Model Checker (https://gander.wustl.edu/~wilson/dmelgenerecord/index.html; Rele et al., 2023), which compares the structure and translated sequence from their hypothesized target gene model against the *D. melanogaster* reference gene model. At least two independent models for each gene are generated by students under mentorship of their faculty course instructors. These models are then reconciled by a third independent researcher mentored by the project leaders to produce a final model like the one presented here. Note: comparison of 5' and 3' UTR sequence information is not included in this GEP CURE protocol.

## Extended Data

Description: GFF, FASTA, PEP. Resource Type: Model. DOI: 10.22002/nn290-0wx55
